# Metabolomics Profiling on Different Stages of Colorectal Cancer: A Systematic Review

**DOI:** 10.21315/mjms2018.25.5.3

**Published:** 2018-10-30

**Authors:** Hazwani Mohd Yusof, Sharaniza Ab-Rahim, Leny Suzana Suddin, Mohd Shahril Ahmad Saman, Musalmah Mazlan

**Affiliations:** Faculty of Medicine, Universiti Teknologi MARA, Jalan Hospital, 47000 Sg Buloh, Selangor, Malaysia

**Keywords:** colorectal cancer, metabolomics, progression, staging, biomarkers

## Abstract

Colorectal cancer (CRC) is one of the leading causes of cancer-related deaths worldwide. Early diagnosis and accurate staging of the disease is vital to improve the prognosis. Metabolomics has been used to identify changes in metabolite profiles in the different stages of cancer in order to introduce new non-invasive molecular tools for staging. In this systematic review, we aim to identify the common metabolite changes in human biological samples and the dominant metabolic pathways associated with CRC progression. A broad systematic search was carried out from selected databases. Four reviewers screened and reviewed the titles, abstracts, and full-text articles according to the inclusion and exclusion criteria. Quality assessment was conducted on the eight articles which met the criteria. Data showed that the metabolites involved with redox status, energy metabolism and intermediates of amino acids, choline and nucleotides metabolism were the most affected during CRC progression. However, there were differences in the levels of individual metabolites detected between the studies, and this might be due to the study population, sample preparation, analytical platforms used and statistical tools. In conclusion, this systematic review highlights the changes in metabolites from early to late stages of CRC. Moreover, biomarkers for prognosis are important to reduce CRC-related mortality.

## Introduction

Colorectal cancer (CRC) is the third most common cancer in men and second in women, with an estimated total number of 1.4 million cases and 693,900 deaths in 2012 (1). The incidence of CRC is several times higher in more developed countries than less developed countries (2). Earlier diagnosis and treatment of CRC has been reported to markedly improve the 5-year survival rate (3).

Improvement in prognosis of CRC has also been suggested to depend on accurate diagnosis and staging of CRC. Currently, the most accepted methods of prognostication are the clinicopathological staging based on the tumour node metastasis (TNM) or the Dukes staging classification systems (4). Colonoscopy in combination with histopathological examination is the current gold standard for diagnosis and staging of CRC. However, the invasive nature and unpleasant clinical procedures, potential risks of complications and relatively poor sensitivity and specificity are the drawbacks of these techniques (5). Therefore, a new non-invasive method is needed. In the quest for new non-invasive CRC detection methods, researchers have turned to metabolomics to identify the molecular phenotypes of CRC.

Metabolomics, the study of small molecular weight metabolites in biological systems (6), has been shown to be useful in distinguishing tumours from healthy tissues and determining the pathophysiology of the disease (7). The changes in metabolomics profiles in diseased versus healthy individuals are not only due to biological alterations but also affected by environmental factors. Metabolomics detects these changes using advanced high-throughput analytical techniques such as nuclear magnetic resonance (NMR), gas chromatography-mass spectrometry (GC-MS) and liquid chromatography-mass spectrometry (LC-MS) together with multivariate statistical analysis (8). In recent years, metabolomics approach has been used to identify tumour-specific biomarkers for cancers, including lung, prostate and breast cancers (9–11). Clinical metabolomics studies of CRC on urine (12, 13), serum (14) and stool (15) samples have provided some potential biomarkers for CRC detection. The metabolomics profiling using tissues of CRC patients has also been analysed to study the process of tumorigenesis, the molecular mechanisms and CRC grading to enhance the accuracy of prognosis, and hence, reduce the CRC-related mortality.

In this systematic review, we analysed and described human studies based on global metabolomics profiling of different stages of CRC. Also, we aimed to identify common metabolomics changes at different stages of CRC as identified by the various studies and discuss the dominant metabolic pathways associated with CRC progression.

## Methods

### Literature Search

A broad systematic search was carried out using PubMed, Web of Science, Scopus and EBSCO*host* (Medline, Cinahl) databases. The searches were conducted using the keywords, such as metabolomic* or metabolome or metabonomic* or metabolite* and colorectal cancer or colon cancer or rectal cancer. Data were searched for up to December 2017. To minimise selection bias, four independent reviewers screened and reviewed the titles, abstracts and full-text articles.

### Inclusion and exclusion criteria

All relevant study designs were included except single case reports, abstracts, posters, proceedings and reviews. The study population involved only human subjects. The target conditions were patients with colorectal cancer, who had not started any medical treatment or surgical interventions. The index test of the selected articles included all types of samples and different stages of colorectal cancer. The reference standard to define the target condition was the histopathological analysis of the resected colorectal cancer specimens. All articles were restricted to the English language.

### Quality assessment

The quality assessment of all the relevant included studies in this review was based on Quality Assessment of Diagnostic Accuracy Studies (QUADAS) tool (16). The QUADAS tool is a 14-question tool, which evaluates the risk of bias for each included study and assesses the quality issues. The evaluations of quality assessment of the included studies were performed by four independent reviewers. Any disagreements were resolved by discussion.

## Results

### Literature Search

**Figure 1:**
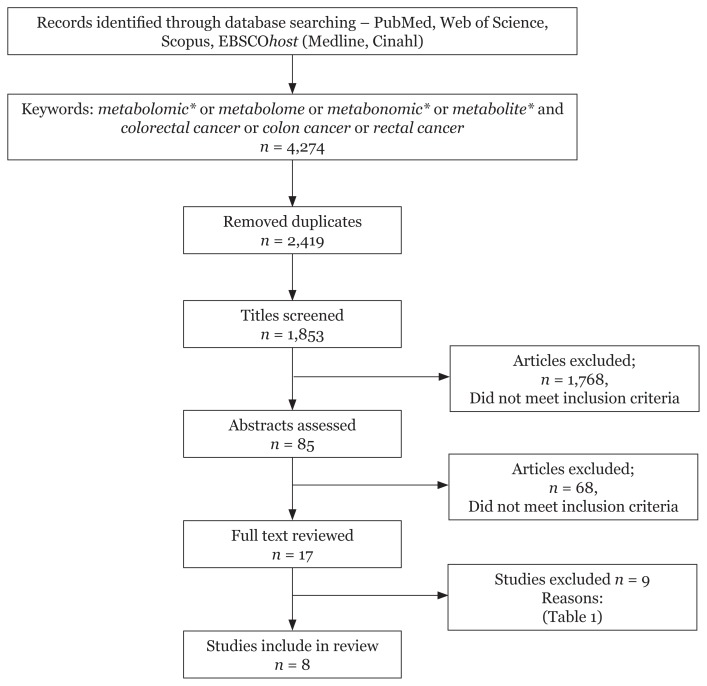
Flow of the selection process

In this systematic review, we used four electronic database searches (PubMed, Web of Science, Scopus and EBSCOhost) and retrieved 4,274 studies for keywords metabolomic* or metabolome or metabonomic* or metabolite* and colorectal cancer or colon cancer or rectal cancer. After removing the duplicate hits, the records were reduced to 1,853 studies. A broad screening of the titles and abstracts was conducted by four independent reviewers and studies which did not meet the inclusion criteria were removed. Further assessment of full articles of the remaining 17 studies resulted in the removal of nine studies due to the reasons listed in Table 1. The remaining eight studies were included in this review. Figure 1 shows the flow of the systematic search carried out in this study.

#### Quality assessment

The results of the quality assessments for each of the included studies are shown in Table 2. All studies included in this review had met the inclusion criteria and most of the items in QUADAS, which indicates that the overall quality of the included studies was good.

#### Descriptions of selected studies

The characteristics of the studies are listed in Table 3. There were eight studies which met the inclusion criteria, three on serum sample (26–28), one on faecal sample (15) and four on tissue sample (29–32). Several analytical platforms were used, including GC-MS, capillary electrophoresis-time-of-flight mass spectrophotometry (CE-TOFMS), ^1^H NMR spectroscopy, high-resolution magic-angle spinning (^1^H HRMAS) NMR spectroscopy and ultra-performance liquid chromatography (UPLC) coupled to TOF ion-mobility spectrometry (TWIMMS).

The study by Nishiumi et al. (26) determined the serum metabolites profile of Japanese CRC patients and healthy volunteers using GC-MS. The study also evaluated the interday and intraday variances of serum metabolites levels. Training set was used to establish the CRC prediction model via multiple logistic regression analysis, and the prediction model was validated using a validation set. CRC was classified into two groups: early CRC consisted of stages 0, 1 and 2 and late CRC included stages 3 and 4 of the disease according to the TNM classification of staging. Mann–Whitney test was used to compare between CRC patients and healthy volunteers. The authors identified 27 metabolites as biomarker candidates.

Vahabi et al. (27) analysed serum of Iranian colon cancer patients from different stages of the disease using NMR. The authors identified the discriminating metabolites using OSC-PLS model and Human Metabolome Database (HMDB), and the affected metabolic pathways using Metaboanalyst Database. The authors showed that the stages of this disease were well separated and identified six metabolites, which can be used to identify the early (stage 0, I) and late (stage II, III, IV) stages. The limitation of this study was the small samples size, as only eight samples per group were analysed.

Uchiyama et al. (28) determined the metabolomics profiles on serum from Japanese CRC patients, healthy controls and colonic adenoma. Samples were collected from a larger pool of an earlier project. In this study, the data were subjected to hierarchical cluster analysis (HCA) and principal component analysis (PCA) using Advanced Human Metabolome Technologies (HMT) Scan package. Statistical analysis compared the metabolomic profiles between CRC, adenoma and healthy subjects. The authors identified 139 known metabolites, which were differently regulated from healthy controls or adenoma patients. Further statistical analysis compared between the different stages of CRC to controls; however, no statistical analysis compared the metabolite levels between each stage. The limitation of the study is the absence of blinding and randomisation during analysis.

Lin et al. (15) undertook a study to identify early CRC biomarkers and metabolic alterations using faecal samples. The study analysed samples from Chinese CRC patients and healthy controls. Metabolomics profiles were obtained using ^1^H NMR spectroscopy coupled to pattern recognition. The Orthogonal Partial Least Squares-Discriminant Analysis (OPLS-DA) was employed to distinguish the metabolomics profiles of CRC from that of the control. The authors also reported that metabolomics profiles showed progressive changes over different CRC stages relative to healthy controls. One of the limitations of this study was diet; the diet of the patients was not considered, which could potentially affect the results.

A tissue metabolomics study was conducted by Wang et al. (29) to determine the metabolomics profiles of rectal tissue from Chinese CRC patients and its adjacent normal-appearing tissues which serve as controls. Samples were analysed using ^1^H NMR. Data were analysed using PCA, PLS-DA and OPLS-DA. The authors identified 40 distinguishing metabolites, of which, 16 were significantly changed during the progression of rectal cancer. The limitations of this study include the absence of blinding or randomisation during analysis and possibility of bias during the selection of samples. On the other hand, the strengths of this study were the large cohort tissue samples, blinded pathological assessment, good description of CRC staging and a detailed data analysis.

Mirnezami et al. (30) developed a sensitive and specific HRMAS NMR metabolic profiling strategy for discriminating the cancerous versus healthy colorectal mucosa, colon versus rectal tumour tissue and tumours of differing T-stage. All tissue samples were collected from London, UK. In this study, 1-dimensional ^1^H Carr-Purcell-Meiboom-Gill (CPMG) spectral profiles from cancer tissues obtained from T1/2 tumours, T3 tumours and T4 tumours were compared to determine the metabolic characteristics utilised for deducing the tumour stage (T-stage). Results for different T-stage showed the OPLS-DA scatter plot could distinguish the metabolic activity among tissue samples obtained from different stages (T1–T2, T3, T4).

Williams et al. (31) used tissue samples from American colon cancer patients to distinguish the metabolomics profiles of cancer tissue with adjacent non-metastatic tissues (NMT) using UPLC coupled to TWIMMS. The authors analysed data using PLS-DA and successfully distinguished the metabolic profiles of CRC tissues and tissues of different stages from NMT. The limitation of this study was the small samples size, especially for stages I and IV. Furthermore, the collection of the control sample was not described in detail.

A metabolomics study on colon and rectal tissues from Chinese CRC patients and their corresponding adjacent non-involved tissue (ANIT) using ^1^H HRMAS NMR was conducted by Tian et al. (32). The study also analysed fatty acid compositions on the same samples using GC-FID/MS. The authors concluded that according to the OPLS-DA strategy, metabolites from the different pathological stages were significantly altered as compared to the ANIT. The metabolites were also differently regulated in the different stages of CRC.

#### Altered metabolites

Most of the studies included in this review were focussed on identifying altered metabolites of CRC as compared to controls and between different cancer stages relative to controls. The studies reported the values of the metabolites at different stages in tables, as box-whisker plots or heat charts in terms of fold-change relative to controls. Tables 4–8 represent the metabolites which were significantly different from the controls according to stages. Upregulation is represented by “+” while “−” denotes downregulation. The numbers denote the fold-change relative to controls.

### Serum Metabolomics Profile

There are three studies on serum global metabolomics analysis using several platforms. Each study analysed their results differently. Nishiumi et al. (26) and Vahabi et al. (27) compared early and late stages of CRC, but their classification of early and late stages differed (Tables 4 and 5).

Nishiumi et al. (26) detected 132 differentiating metabolites, but only reported the 27 metabolites which met their criteria as biomarker candidates (RSD % value of < 20%; not significant (*P* ≥ 0.05) intraday or interday variances and a significant difference (*P* < 0.05) between CRC patients and healthy volunteers). Table 4 showed the fold-change of each selected metabolites at early (0–II) and late (III–IV) stages of the disease as compared to healthy volunteers.

Vahabi et al. (27) identified six differentiating metabolites from the two groups: deoxydinose, pyroxidine, glycine, taurocholic acid, cholesteryl ester, deoxycholic acid (Table 5). Five of these metabolites were decreased in the early stage (0, I), but increased in the late stage (II, III, IV). One metabolite, pyroxidine, was increased in the early stage and decreased in the late stage.

Uchiyama et al. (28) reported the metabolite profiles as the ratio of the metabolites to healthy controls in different CRC stages (Table 6). The data showed that the metabolites were differentially regulated at different stages of CRC relative to controls. However, 1-methylnicotinamide, 2-aminoisobutyric acid, 3-hydroxybutyric acid, citric acid, decanoic acid, gluconoic acid, glutamate, hypoxanthine, inosine, o-acetylcarnitine, octanoic acid and uridine remained elevated throughout the different stages of CRC while 3-indoxylsulfuric acid, alanine, arginine, asparagine, benzoic acid, betaine, butyrate, cholic acid, choline, citrulline, ethanolamine, ethanolamine phosphate, glucoronic acid, glycerol 3-phosphate, histidine, homovanillic acid, hydroproline, indolE-3-acetic acid, isobutyric acid, isovaleric acid, lauric acid, lysine, methionine, N^2^-phenylacetylglutamine, ornithine, phenylalanine, pipecolic, proline, quinic acid, sarcosine, succinate, threonic acid, trimethylamine N*-*oxide, tryptophan, tyrosine, β-alanine and γ-butyrobetaine were downregulated.

Based on the correlation studies and further analysis of areas under the receiver operating characteristic (ROC) curves (AUC), the authors concluded that benzoic acid, octanoic acid, decanoic acid and histidine were significantly correlated with CRC stages. They also reported that benzoic acid was most significantly correlated with staging.

### Faecal Metabolomics Profiles

Lin et al. (15) reported their findings in terms of relative intensity of the metabolites in CRC samples and normal healthy individuals. The study identified 14 metabolites in CRC which were regulated differently than the healthy controls. Alanine, dimethylglycine, glutamate, isoleucine, lactate, leucine, proline, succinate and valine were upregulated throughout stages I to IV of CRC, while acetate, butyrate, glucose, glutamine and propionate were downregulated. The group further reported the differences in the relative intensities of metabolites at each stage of CRC as compared to healthy controls in box-and-whisker plots (Table 6). Based on the box-and-whisker plots, the authors showed progressive changes in the metabolite levels over different CRC stages relative to healthy controls, although no statistically significant values were reported. The authors concluded that faecal metabolites can be used to distinguish CRC from healthy controls, and acetate and succinate were termed as the best candidates for biomarkers.

### Tissue Metabolomics Profiles

Wang et al. (29) analysed the metabolite profiles on rectal tissues of CRC and compared to the normal mucosa of the same patients. The study identified 37 metabolites that were significantly different from the control. Also, they compared the metabolite profiles of the different stages of CRC and, these were tabulated in terms of fold-change of intensity of metabolites at different stages relative to controls (Table 7). Betaine, creatine, dimethylglycine, glucose, glyceryl, glycolate, mannitol, myo-inositol, phosphocreatine, taurine and tyrosine were downregulated throughout stages I to IV of CRC. Alanine, β-hydroxybutyrate, lactate, phosphorylcholine and trimethylamine N-oxide were upregulated in rectal cancer from stages II to IV. Acetate, dimethylamine, leucine, glutamine and O-acetylglycoprotein were significantly altered in stages III and IV, while acetone, acetoacetate, formic acid, isoleucine, NAD, sarcosine and valine were upregulated only in stage IV. The authors concluded that glycolysis, tricarboxylic acid cycle (TCA), choline metabolism, ketone bodies and amino acid metabolisms are the most affected pathways.

Mirnezami et al. (30) have determined a total of 171 CPMG spectral profiles obtained from cancer tissues and healthy controls. After determination of metabolic differences between stages, they found that the T3 tumour tissue had significantly greater concentrations of lipids/ triglycerides (*P* < 0.05) and acetate (*P* < 0.05) than T1/2 tumours, whereas the T1/2 tumours contained higher levels of glycerophosphoryl-choline (*P* < 0.05) than T3 tumours. Moreover, T4 showed significantly reduced levels of lipids/ triglycerides, acetate and succinate relative to the T3 tumours. These metabolite fluxes are summarised in Table 8.

The relative intensity of metabolites at each stage of cancer was compared to NMT and reported in terms of heat maps by Williams et al. (31). These values were tabulated in Table 7 in terms of fold-change relative to NMT. The results showed that the metabolites that can be used as biomarkers of CRC include nucleotides, nucleosides, bile acids, and oxidative metabolites. Furthermore, the authors and analysed their data to identify the biomarkers which can differentiate the four stages of CRC. They observed that levels of (S)-2-acetolactate, 1,3-dimethyluric acid, 1,9-dimethyluric acid, 3′-UMP, adrenochrome o-semiquinone, arachidonic acid, conjugated linoleic acid, ethyl 9-hexadecenoate, ethylmalonic acid, γ-glutamyl-β-cyanoalanine, glutathione, glyceryl phosphorylethanolamine, inosine, inositol cyclic phosphate, isoleucine, leucine, L-glutamic γ-semialdehyde, myristic acid, N-acetyl-glucosamine 1-phosphate, palmitic acid, stearic acid, taurine, tyrosine, vaccenic acid and xanthine were elevated at all stages of the disease. However, they also observed that docosahexaenoic acid, glucose 1-phosphate and hypoxanthine were upregulated initially but downregulated in the later stage CRC as compared to the earlier stage. Notably, the number of samples for each stage was not equal as there were more stage III samples and that there was only one sample each for stages I and IV, respectively.

Tian et al. (32) reported that the amounts of alanine, aspartate, choline, cysteine, cytosine, glutamate, glutamine, glutathione, glycerophosphocholine, glycine, isocytosine, isoleucine lactate, leucine, phenylalanine, phosphoethanolamine, phosphorylcholine, scyllo-inositol, taurine, tyrosine, uracil and valine were higher in CRC samples as compared to ANIT. Sarcosine level was increased at the later stage of CRC, while the lipid levels in CRC were found to be significantly lower than ANIT.

The authors (32) also observed that stage I had the largest difference in metabolite profiles between CRC and ANIT, which decreased in the high-grade samples except for lactate (Table 7). Although this study could not distinguish the metabolite profiles between stages I and II and stages III and IV, it demonstrated that the metabolite levels in stages I–II are different from stages III–IV. In terms of lipid metabolites, the authors reported that the levels of oleic acid, eicosenoic acid, linoleic acid, eicosadienoic acid and α-linoleic acid were lower in stages I–II, while in stage III–IV, only eicosenoic acid was lower than ANIT. The study also found a higher level of eicosadienoic acid in stages III–IV as compared to stages I–II.

## Discussion

This systematic review was conducted to summarise the metabolomics profiles of CRC human biological samples associated with its progression. Although there are many metabolomics studies on CRC, the data on global metabolomics changes in different stages of CRC is limited. Thus, in this systematic review, we discussed the findings from eight studies that met the inclusion and exclusion criteria.

### Serum Metabolomics Profile

Nishiumi et al. (26) identified 27 metabolomics as CRC biomarker candidates. However, these metabolites displayed individual AUC values of 0.6 to −0.8 and relatively low sensitivity or specificity. Thus, single metabolites biomarkers are not practical for disease screening or diagnosis. Furthermore, Nishiumi et al. (26) and Uchiyama et al. (28) identified similar differentiating metabolites, such as β-alanine, ribulose, asparagine, ornithine, citrulline, kynurenine and cystine. However, the levels of these metabolites differed between the two studies, although both studies were on the same population, i.e., Japanese.

Vahabi et al. (27) reported that the level of glycine increased in the late stage of CRC; a similar observation was reported by Uchiyama et al. (28). Glycine is a vital amino acid and biosynthetically linked with serine. It provides the essential precursors for the synthesis of proteins, nucleic acids and lipids that are crucial for cancer cell growth (33). Also, the uptake and catabolism of glycine can promote tumorigenesis and malignancy, suggesting that glycine metabolism could be a target for therapeutic intervention (34).

Uchiyama et al. (28) reported 139 known metabolites, and of these, only 24 were highly correlated with CRC stages. Consequently, the authors suggested benzoic acid as the best biomarker to detect the CRC; the level of benzoic acid decreased along with the progression of CRC. Interestingly, previous studies on CRC serum metabolomics analysis did not identify benzoic acid as a potential biomarker (35–38). This is probably due to the difference in the study population and analytical platforms used. CE-MS analysis can detect highly polar and charged metabolites. Benzoic acid is produced by the human gut microbiota from degradation of procyanidins (39), and hence, its reduced level in CRC may reflect the alterations in the normal gut microbiota.

### Faecal Metabolomics Profiles

In the study using faecal samples, the authors reported that faecal metabolites of CRC patients at the early stage were significantly distinct from healthy controls with acetate and succinate as the best candidates for biomarkers. The alterations in the metabolomic profiles may be the result of changes in the normal bacterial ecology, malabsorption of nutrients or altered metabolisms, which might lead to the initiation and the progression of CRC (15).

Lipid metabolites of acetate, butyrate and propionate were shown to be downregulated at all stages of CRC, while succinate was upregulated. Acetate and succinate were not only found in faecal metabolomics study but also observed in tissues by Mirzenami et al. (30). Acetate and butyrate are short chain fatty acids (SCFAs) providing energy to the intestinal cell wall (40). A decreased level of these metabolites might be caused by the disruption of intestinal microbiota and host tissue associated with colorectal tumorigenesis (15). Gut microbiota in human intestine affects the metabolism and signalling pathways of the hosts, especially those involved in the digestion of unutilised energy substrates to produce important biological metabolites such SCFAs (41).

Succinate is a tricarboxylic acid (TCA) cycle metabolite, and defects in the TCA cycle have been reported to contribute to tumour formation. The accumulation of succinate may be due to the downregulation of succinate dehydrogenase (SDH), which then transmits an “oncogenic” signal from mitochondria to the cytosol. Increased succinate level has also been suggested to increase the expression of genes involved in angiogenesis, metastasis and glycolysis, leading to tumour progression (42).

As shown in Table 6, glucose and lactate were observed to be downregulated and upregulated respectively, throughout CRC progression. These findings were as expected and are due to the Warburg effect observed in cancer cells (43). Several amino acids were observed to remain upregulated throughout the different stages of CRC. Glutamine is the only amino acid that was found to be downregulated. The alterations in amino acid profiles could be caused by malabsorption of nutrients due to epithelium inflammation and injury resulting from a bowel disease (15). Notably, the proline level in faeces was affected as compared to other types of samples. Thus, faecal proline serves as an exfoliated marker and is derived from the shedding of colonocytes at the gut luminal surface (44).

#### Tissues metabolomics profiles

All the studies included in this review reported the ability to identify the metabolites which might serve as biomarkers of CRC. They also showed that the levels of some metabolites are changed between the different stages of CRC. However, there are differences in the metabolite profiles reported. These differences could be due to the differences in the study population, tissue samples, sample preparation, analytical platforms and statistical analyses used. The type of tissue samples also differed. Mirnezami et al. (30) and Tian et al. (32) analysed both colon and rectal tissues, while Wang et al. (29) analysed only rectal tissues and Williams et al. (31) analysed the colon tissues.

Three studies in this review used ^1^H NMR analysis, and only one study by Williams et al. (31) used UPLC. In addition, Tian et al. (32) used GC-FID/MS to identify the fatty acid profiles, thereby reporting a larger number of lipid metabolites same as the study by Williams et al. (31) which used UPLC; as a result, a larger number of lipid and nucleotide metabolites were detected. UPLC/MS preferentially detects hydrophobic molecules, while ^1^H NMR detects small molecular weight molecules which are present in high concentrations (45).

The data from Tables 4, 5 and 6 showed the common metabolite changes reported in the serum metabolomics studies (26–28). These altered metabolites were those that are involved in redox status, energy, amino acid, choline and nucleotides metabolisms. The alterations indicate possibility of disturbances in the associated metabolic pathways as CRC progresses. Glutathione, isoleucine and leucine were reported by three studies to remain upregulated along with CRC progression. Alanine, glutamine, glycerophosphocholine, lactate, phosphorylcholine, sarcosine, uracil and valine were reported to be upregulated by two of the four studies. In contrast, glycerophosphocholine level was reported to decrease from stage T1-T2 to T3 in Minerzami et al. (30). Taurine and tyrosine were reported to be upregulated by Williams et al. (31) and Tian et al. (32) but downregulated in a study by Wang et al. (29).

Glutathione is a major biomolecule for cellular protection against oxidative stress and detoxification of xenobiotics. The high level of glutathione in cancer tissues is correlated with a proliferative response and essential for cell cycle progression (46). Leucine, isoleucine and valine are required for protein synthesis (47), and the increased level of these amino acids may reflect the cellular needs for protein for continuous growth and proliferation of cancer cells (31). Glutamine provides nitrogen for cell growth and proliferation, and in addition, it acts as a carbon source for mitochondrial metabolism (48, 49).

The change in the levels of lactate is consistent with the Warburg effect associated with cancer cells (43). Although Warburg attributed this to mitochondrial damage, later studies showed that the mitochondria of most tumour cells are functional and that the tumour cells use oxidative phosphorylation and glycolysis for cell growth (50, 51). Ward and Thompson (51) suggested that in proliferating cells, mitochondrial metabolism is re-programmed to support anabolic pathways. They further suggested that utilising mitochondrial oxidative phosphorylation in cancer cells to produce energy is secondary to glycolytic pathway. The glycolytic pathway has been suggested as crucial for cancer cells as its intermediates are used for anabolic reactions; for example, the synthesis of glycogen and pentose phosphates from glucose 6-phosphate (52).

Wang et al. (29) and Tian et al. (32) reported that the levels of phosphorylcholine, glycerophosphocholine and sarcosine were upregulated as compared to normal. These metabolites are involved in the choline metabolism pathway, which provides substrates for phospholipid metabolism of cell membranes; they are also identified as markers of cell proliferation (29).

Uracil was noted to be upregulated by Williams et al. (31) and Tian et al. (32). Uracil is a demethylated form of thymine, a nucleobase in DNA and plays critical roles in DNA stability and replication. The increased level of uracil in CRC tumours might be due to a decline in the dihydropyrimidine dehydrogenase (DPD) activity (53).

The metabolites profiles reported by four different studies on tissues differed due to the heterogeneity of study populations and methods employed, which restricted the final conclusions obtained from these data. Furthermore, the number of studies included was also small to draw a valuable conclusion. However, this review highlighted the lack of data on metabolomics profiles in the different stages of CRC. Thus, these data would aid our understanding of the progression of the disease as well as identifying biomarkers and improving prognosis.

## Conclusions

In conclusion, metabolic changes during the progression of CRC can be identified using metabolomics approach. Glutathione, isoleucine and leucine were upregulated in all stages of CRC but could not be differentiated between the stages. However, the numbers of studies reviewed are small due to the lack of published data on this aspect. As metabolomics profile is affected not only by biological changes but by the diet, lifestyle, medication, chronic diseases and environmental exposure, more studies are needed in this field.

### Future Perspectives

The lack of data on metabolomics profiles during CRC stages indicates the need for additional studies in this field. The identification of biomarkers which can differentiate the different stages of CRC as well as understanding the pathophysiology of CRC progression will help in the treatment strategies and improve the prognosis of the disease. As metabolomics profiles are influenced by environmental factors, the analytical platforms used, samples and methods of sample extraction and data analyses should be standardised for easy comparison among the studies.

## Figures and Tables

**Table 1:** Excluded studies

Study	Reason for Exclusion
Bedin et al. (17)	Proteomics study
Chen et al. (18)	Analysis did not take into account the different stages
Farshidfar et al. (19)	Result did not include list of metabolites of different stages
Jimenez et al. (20)	Sampling method unclear
Qiu et al. (21)	Study did not involve CRC progression
Aleksandrova et al. (22)	This is a longitudinal cohort study
Wang et al. (23)	Study only involved in early CRC
Jordan et al. (24)	Study did not involve in different stages of CRC
Lin et al. (25)	Article in Chinese

**Table 2:** Quality assessment of included studies

	Description/Article	1	2	3	4	5	6	7	8
1	Was the spectrum of patient’s representative of the patients who will receive the test in practice?	+	+	+	+	+	+	+	+
2	Were selection criteria clearly described?	+	+	+	?	?	+	+	+
3	Is the reference standard likely to correctly classify the target condition?	+	+	+	+	?	+	+	+
4	Is the time period between reference standard and index test short enough to be reasonably sure that the target condition did not change between the two tests?	?	?	+	+	?	+	+	?
5	Did the whole sample of a random selection of the sample, receive verification using a reference standard of diagnosis?	+	+	+	+	+	+	+	?
6	Did patients receive the same reference standard regardless of the index test results?	+	+	+	?	?	+	+	?
7	Was the reference standard independent of the index test (i.e., the index text did not form part of the reference standard)?	+	+	+	+	?	?	+	+
8	Was the execution of the index test described in sufficient detail to permit replication of the test?	+	+	+	+	+	+	+	+
9	Was the execution of the reference standard described in sufficient detail to permit its replication?	+	+	+	+	?	+	+	+
10	Were the index tests results interpreted without knowledge of the results of the reference standard?	−	−	−	−	−	−	−	−
11	Were the reference standard results interpreted without knowledge of the results of the index test?	−	−	−	−	?	−	−	−
12	Were the same clinical data available when test results were interpreted as would be available when the test is used in practice?	+	+	+	+	+	+	+	+
13	Were uninterpretable/intermediate test results reported?	+	+	−	−	−	−	−	+
14	Were withdrawals from the study explained?	−	+	?	−	−	−	−	+

Risk of bias summary: review authors’ judgements about each risk of bias item for each included study. +: Yes (high quality), −: No (low quality), ?: Unclear

1: Nishiumi et al. (26), 2: Wang et al. (29), 3: Minerzami et al. (30), 4: Williams et al. (31), 5: Vahabi et al. (27), 6: Lin et al. (15), 7: Tian et al. (32), 8: Uchiyama et al. (28)

**Table 3:** Characteristics of included studies

	Study	Platform	Study design	Sample Extraction	Type of samples	Sample size
1	Nishiumi et al. (26)	GC/MS	Case series	Extraction buffer: Methanol Water Chloroform	Serum	Training set CRC patients *n* = 60 • Stage 0: 12, Stage I: 12, Stage II: 12, Stage III: 12, Stage IV: 12 Control: • Healthy control *n* = 60 Validation set • CRC patients *n* = 59 • Stage 0: 15, Stage I: 11, Stage II: 13, Stage III: 11, Stage IV: 19 Control: • Healthy control *n* = 63
2	Vahabi et al. (27)	^1^H NMR	Case series	Extraction buffer: Deuterated water	Serum	CRC patients *n* = 16 • Stage 0–I: 8, Stage II–IV: 8
3	Uchiyama et al. (28)	CE-TOFMS	Case series	Extraction buffer: Methanol Chloroform	Serum	CRC patients *n* = 56 • Stage I: 14, Stage II: 14, Stage III: 14, Stage IV: 14 Control: • Healthy control *n* = 59
4	Lin et al. (15)	^1^H NMR	Case series	No extraction	Faecal	100 fecal sample • 68 CRC, Stage I/II: 20, Stage III: 25, Stage IV: 23 • 32 healthy
5	Wang et al. (29)	^1^H NMR	Case series	Extraction buffer: Methanol Chloroform	Colon Tissue	CRC patients *n* = 127 • Stage I: 35, Stage II: 37, Stage III: 37, Stage IV: 18 Control: • Adjacent non-tumour tissue *n* = 43
6	Mirnezami et al. (30)	HR-MAS NMR	Prospective observational study	Extraction buffer: Deuterated water	Colorectal mucosa	CRC patients *n* = 44 • Stage I/II: 12, Stage III: 20 • Stage IV: 12
7	Williams et al. (31)	UPLC TWIMMS	Case series	Extraction buffer: Acetonitrile Water Formic acid	Rectal Tissue	CRC patients *n* = 9 • Stage I: 1, Stage II: 3, Stage III: 5, Stage IV: 1 Control: • Adjacent non-tumour tissue *n* = 9
8	Tian et al. (32)	^1^H HRMAS NMR GC-FID/MS (analysed the fatty acid)	Case series	Extraction buffer: Deuterium oxide Methanol	Colon and Rectal Tissue	CRC patients *n* = 50: • 16 colon cancer • 34 rectal cancer Stage I: 16, Stage II: 12, Stage III: 17, Stage IV: 5 Control: • Adjacent non-involve tissue (ANIT) *n* = 50

**Table 4:** Differentiating serum metabolites between early and late stages of CRC [Nishiumi et al. (26)]

Metabolites	Stages	

	0–II	III–IV
2-hydroxy-butyrate	1.59	2.18
Arabinose	1.61	1.71
Asparagine	1.22	1.10
Aspartic acid	1.52	1.69
Citrulline	1.16	1.14
Creatinine	0.78	0.82
Cystamine	1.57	1.21
Cystine	1.81	1.16
Glucosamine_2	1.12	1.13
Glucuronate_1	1.12	1.23
Glutamic acid	1.54	2.24
Inositol	1.19	1.17
Isoleucine	1.39	1.29
Kynurenine	1.74	1.96
Lactitol	1.16	16.87
Meso-erythritol	3.01	2.57
Nonanoic acid (C9)	0.71	0.67
O-phosphoethanolamine	0.89	0.94
Ornithine	1.29	1.21
Palmitoleate	1.31	1.20
Phosphate	1.10	1.30
p-hyrdroxybenzoic acid	1.89	1.58
Pyroglutamic acid	1.44	1.15
Pyruvate	1.21	1.56
Ribulose	0.65	0.89
Xylitol	1.22	1.51
β-alanine	1.30	1.25

Fold change of each selected metabolite as biomarker candidates in the CRC patients with stage 0–2 and stage 3–4 disease compared with healthy volunteers. Data is from the training set

**Table 5:** Differentiating serum metabolites separating the early and late stages of CRC [Vahabi et al. (27)]

Metabolites	Stages	

	0–1	II–IV
Cholesteryl ester [18:2(9z,12z)]	−3	+3
Deoxycholic acid	−	+
Deoxydinosine	−	+
Glycine	−	+
Pyroxidine	+	−
Taurochoilic acid	−2	+

−: downregulated, +: upregulated, 0: no change detected

**Table 6:** Metabolomics profile in different stage of CRC using serum and fecal samples

Samples	Serum				Fecal			
		
Studies	Uchiyama et al. (28)				Lin et al. (15)			
		
Stages	I	II	III	IV	I	II	III	IV
		
Metabolites								
10-Hydroxydecanoic acid	+	+	0	−				
1-Methylnicotinamide	−	+2	+	+2				
2-Aminoisobutyric acid	+	+	+	+				
3-Hydroxybutyric acid	+4	+5	+5	+2				
3-Indoxylsulfuric acid	−	−	−	−				
3-Methylhistidine	−	−	−	−				
4-Methyl-2-oxovaleric acid	+	−	−	−				
Acetate					−	−	−	−
Alanine	−	−	−	−	+	+	+	+
Arginine	−	−	−	−				
Ascorbate 2-sulfate	0	0	−	+				
Asparagine	−	−	−	−				
Benzoic acid	−	−	−	−				
Betaine	−	−	−	−				
Butyrate	−	−	−	−	−	−	−	−
Cholic acid	0	−	−	−				
Choline	−	−	−	−				
Cis-aconitic acid	+	+	0	0				
Citric Acid	+	+	+	+				
Citrulline	−	−	−	−				
Cysteine glutathione disulfide	0	−	−	0				
Cystine	0	0	−	−				
Decanoic acid	+13	+9	+12	+7				
Diethanolamine	0	+	−	−				
Dimethylglycine					+	+	+	+
Ethanolamine	−	−	−	−				
Ethanolamine phosphate	−	−	−	−				
Gluconoic acid	+3	+7	+12	+9				
Glucoronic acid	−	−	−	−				
Glucose					−	−	−	−
Glutamate	+	+	+	+	+	+	+	+
Glutamate-glycine	0	+	+	−				
Glutamine	−	0	−	−	−	−	−	−
Glycerol 3-phosphate	−	−	−	−				
Glycine	−	0	0	0				
Glycine-threonine	+	+	0	+				
Heptanoic acid	0	0	0	+				
Hippuric acid	−	−	−	+				
Histidine	−	−	−	−				
Homovanillic acid	−	−	−	−				
Hydroxyproline	−	−	−	−				
Hypoxanthine	0	+	+	+				
IndolE-3-acetic acid	−	−	−	−				
Inosine	+	+	+	+				
Isethionic acid	−	+	−	0				
Isobutyric acid	−	−	−	−				
Isobutyryl carnithine	0	0	+2	−				
Isocitric acid	+	+	0	0				
Isoleucine					+	+	+	+
Isovaleric acid	−	−	−	−				
Kynurenine	−	−	0	+				
Lactate					+	+	+	+
Lauric acid	−	−	−	−				
Leucine	0	0	−	−	+	+	+	+
Lysine	−	−	−	−				
Methionine	−	−	−	−				
Mucic acid	+	+	0	+				
Myristoleic acid	+	0	−	−				
N^2^-Phenylacetylglutamine	−	−	−	−				
*N5*−Ethylglutamine	+	−	+	+				
O-Acetylcarnitine	+	+	+	+				
Octanoic Acid	+32	+31	+30	+18				
Ornithine	−	−	−	−				
Perillic Acid	+	−	−	−				
Phenylalanine	−	−	−	−				
Pipecolic	−	−	−	−				
Proline	−	−	−	−	+	+	+	+
Propionate					−	−	−	−
Quinic acid	−	−	−	−				
Ribulose 5 -phosphate	−	+	+	−				
Sarcosine	−	−	−	−				
*S*-Methylcysteine	−	−	0	−				
Stachydrine	+	−	−	−				
Succinate	−	−	−	−	+	+	+	+
Taurine	0	0	−	0				
Threonic acid	−	−	−	−				
Threonine	−	−	−	0				
Trimethylamine N*-*Oxide	−	−	−	−				
Tryptophan	−	−	−	−				
Tyrosine	−	−	−	−				
Urea	−	0	−	0				
Uric acid	−	0	−	−				
Uridine	0	+	+	+				
Valine	0	−	−	−	+	+	+	+
β-Alanine	−	−	−	−				
γ-Butyrobetaine	−	−	−	−				

−: downregulated, +: upregulated, 0: no change detected

Numericals denote fold change (FC) relative to control and was assigned as follows:

+ or − : 0.1–1.0 FC

2 : 1.1–2.0 FC

3 : 2.1–3.0 FC

4 : 3.1–4.0 FC

5 : 4.1–5.0 FC

**Table 7:** Metabolomics profile in different stages of CRC using tissue

Studies	Wang et al. (29)				Williams et al. (31)				Tian et al. (32)			
			
Stages	I	II	III	IV	I	II	III	IV	I	II	III	IV
			
Metabolites												
(S)-2-Acetolactate					+	+	+	+				
1,3-Dimethyluric acid					+4	+3	+2	+4				
1,9-Dimethyluric acid					+4	+3	+2	+4				
2-Hydroxylbutyrate acid	−2	−2	−2	0								
3′-UMP					+	+	+	+				
Acetate	0	0	+7	+3								
Acetoacetate	0	0	0	+2								
Acetone	0	0	0	+2								
Adenine					+	+2	0	+2				
Adrenochrome- o-semiquinone					+	+	+	+				
Alanine	0	+2	+2	+2					+	+	+	+
Arabinosylhypoxanthine					0	+2	+	0				
Arachidonic acid					+	+	+2	+				
Aspartate									+2	+2	+2	+
Betaine	−2	−3	−2	−2								
Choline									+3	+2	+	+
Conjugated linoleic acid					+2	+3	+3	+				
Creatine	−3	−3	−3	−3								
Cysteine									+3	+2	+	+
Cytidine					+	+	0	+				
Cytidine monophsphate					+	+	0	+				
Cytosine									+2	+	+	+
Deoxycholic acid					+	0	+	0				
Deoxyribose					+	0	0	+				
Dimethylamine	0	0	+2	+3								
Dimethylglycine	−3	−3	−3	−3								
Docosahexaenoic acid					+	+	+	0				
Ethyl 9-hexadecenoate					+2	+	+	+				
Ethylmalonic acid					+	+	+	+				
Formic acid	0	0	0	+2								
Fructose 2,6-bisphosphate					0	+	+	0				
γ-Glutamyl-β-cyonoalanine					+	+	+2	+				
Glucose	−2	−2	−3	−3								
Glucose 1-phosphate					+	+	+	0				
Glutamate									+3	+3	+2	+2
Glutamine	0	0	+2	+2					+2	+	+	+
Glutathione	+	+2	+2	+2	+3	+3	+3	+3	+3	+3	+	+
Glyerophosphoinositol					0	+	+	0				
Glycerophosphocholine	+2	+2	+2	+2					+2	+2	+2	+
Glyceryl	−2	−2	−2	−2								
Glycerylphosphorylethanolamine					+	+	+	+				
Glycine									+2	+2	+2	+2
Glycolate	−3	−3	−3	−3								
β-Hydroxybutyrate	−	+3	+3	+2								
Hypoxanthine					+	+	++	0				
Inosine					+4	+3	+3	+4				
Inositol cyclic phosphate					+	+	+	+				
Isocytosine									+3	+	+	+
Isoleucine	0	0	0	+2	+	+	+	+	+	+	+	+
Lactate	0	+2	+2	+2					+2	+2	+2	+3
Leucine	0	0	+2	+2	+	+	+	+	+	+	+	+
L-Glutamatic γ-semialdehyde					+	+	+	+				
Lipid									−	−	−	−
Lysine	0	+2	+2	0								
Mannitol	−2	−2	−2	−2								
Myo-inositol	−2	−2	−2	−2								
Myristic acid					+	+	+	+				
N-Acetyl-9-O-acetylneuraminic acid					+	+	0	+				
N-Acetyl-glucosamine1-phosphate					+	+	+	+				
NAD	0	0	0	+3								
O-Acetyhlglycoprotein	0	0	+3	+2								
Palmitic acid					+3	+4	+4	+2				
Phenylalanine									+	+2	+	+2
Phosphocreatine	−4	−3	−2	−2								
Phosphoethanolamine									+2	+2	+	+
Phosphorycholine	0	+2	+2	+2					+3	+2	+2	+
Sarcosine	0	0	0	+3							+	+
*Scyllo*-inositol									+2	+	+	+2
Serine	+2	+2	+2	+2								
Stearic acid					+2	+	+	+				
Succinate	+2	+2	+2	+2								
Taurine	−2	−2	−3	−3	+2	+3	+3	+	+5	+4	+2	+3
Threonine	+2	+2	+2	+2								
Trimethylamine N-oxide	0	+4	+3	+3								
Trymethyltridecanoic acid					0	+	+	0				
Tryptophan	−2	−2	0	0								
Tyrosine	−2	−2	−2	−2	+	+	+	+	+2	+2	+2	+2
Uracil	+3	+4	+3	+3					+4	+3	+2	+2
Vaccenic acid					+2	+4	+4	+				
Valine	0	0	0	+4					+	+2	+	+
Xanthine					+	+	+	+				

−: downregulated, +: upregulated, 0: no change detected

Numericals denote fold change (FC) relative to control and was assigned as follows:

+ or − : 0.1–1.0 FC

2 : 1.1–2.0 FC

3 : 2.1–3.0 FC

4 : 3.1–4.0 FC

5 : 4.1–5.0 FC

**Table 8:** Differentiating tissue metabolites between early and late stages of CRC [Mirnezami et al. (30)]

Metabolites	Stages	

	T1/2 vs T3	T3 vs T4
Acetate	+	−
Glycerophosphorylcholine (GPC)	−	0
Lipids/triglycerides	+	−
Succinate	0	−

−: downregulated, +: upregulated, 0: no change detected
